# Effects of percutaneous coronary intervention on dyspnea in stable coronary artery disease

**DOI:** 10.1007/s00392-022-02107-x

**Published:** 2022-09-13

**Authors:** Michael Wester, Franziska Koll, Mark Luedde, Christoph Langer, Markus Resch, Andreas Luchner, Karolina Müller, Florian Zeman, Michael Koller, Lars S. Maier, Samuel Sossalla

**Affiliations:** 1 Department of Internal Medicine II, University Heart Centre Regensburg, University Medical Center Regensburg, University Hospital Regensburg, Franz-Josef-Strauß-Allee 11, 93053 Regensburg, Germany; 2 Kardiologische Gemeinschaftspraxis Bremerhaven, Bremerhaven, Germany; 3 Kardiologisch-Angiologische Praxis, Heart Centre Bremen, Bremen, Germany; 4 Department of Internal Medicine I, St. Josef Hospital, Regensburg, Germany; 5 Department of Cardiology, Hospital Barmherzige Brüder Regensburg, Regensburg, Germany; 6 Centre for Clinical Studies, University Hospital Regensburg, Regensburg, Germany

**Keywords:** PCI, Stable CAD, QoL, PLA-pCi-EBO, Dyspnea

## Abstract

**Background:**

Dyspnea is a frequent symptom in patients with stable coronary artery disease (CAD) and is recognized as a possible angina equivalent.

**Objectives:**

This study was to assess the impact of percutaneous coronary intervention (PCI) on dyspnea, quality of life, and angina pectoris in patients with stable CAD.

**Methods:**

The prospective, multi-center PLA-pCi-EBO-pilot trial included 144 patients with symptomatic stable CAD and successful PCI. The prespecified endpoints angina pectoris (Seattle Angina Questionnaire–SAQ) and dyspnea (NYHA scale) were assessed 6 months after PCI. Predictors for symptomatic improvement were assessed with uni- and multivariable logistic regression analyses.

**Results:**

Patients with concomitant dyspnea had worse SAQ physical limitation scores at baseline (49.5 ± 21.0 vs 58.9 ± 22.0, *p = *0.013) but showed no difference for angina frequency or quality of life. Overall, symptomatic burden of angina pectoris and dyspnea was alleviated by PCI. However, patients with concomitant dyspnea had markedly worse scores for physical limitation (78.9 ± 25.0 vs 94.3 ± 10.6, *p < *0.001), angina frequency (77.9 ± 22.8 vs 91.1 ± 12.4, *p < *0.001), and quality of life (69.4 ± 24.1 vs 82.5 ± 14.4, *p < *0.001) after PCI. The prevalence of dyspnea (NYHA class ≥ 2) declined from 73% before PCI to 54%. Of 95 initially dyspneic patients, 57 (60%) improved at least one NYHA class 6 months after PCI. In a multivariable logistic regression analysis, “atypical angina pectoris” was associated with improved NYHA class, whereas “diabetes mellitus” had a negative association.

**Conclusion:**

PCI effectively reduced dyspnea, which is a frequent and demanding symptom in patients with CAD. The German Clinical Trials Register registration number is DRKS0001752 (www.drks.de).

**Graphical abstract:**

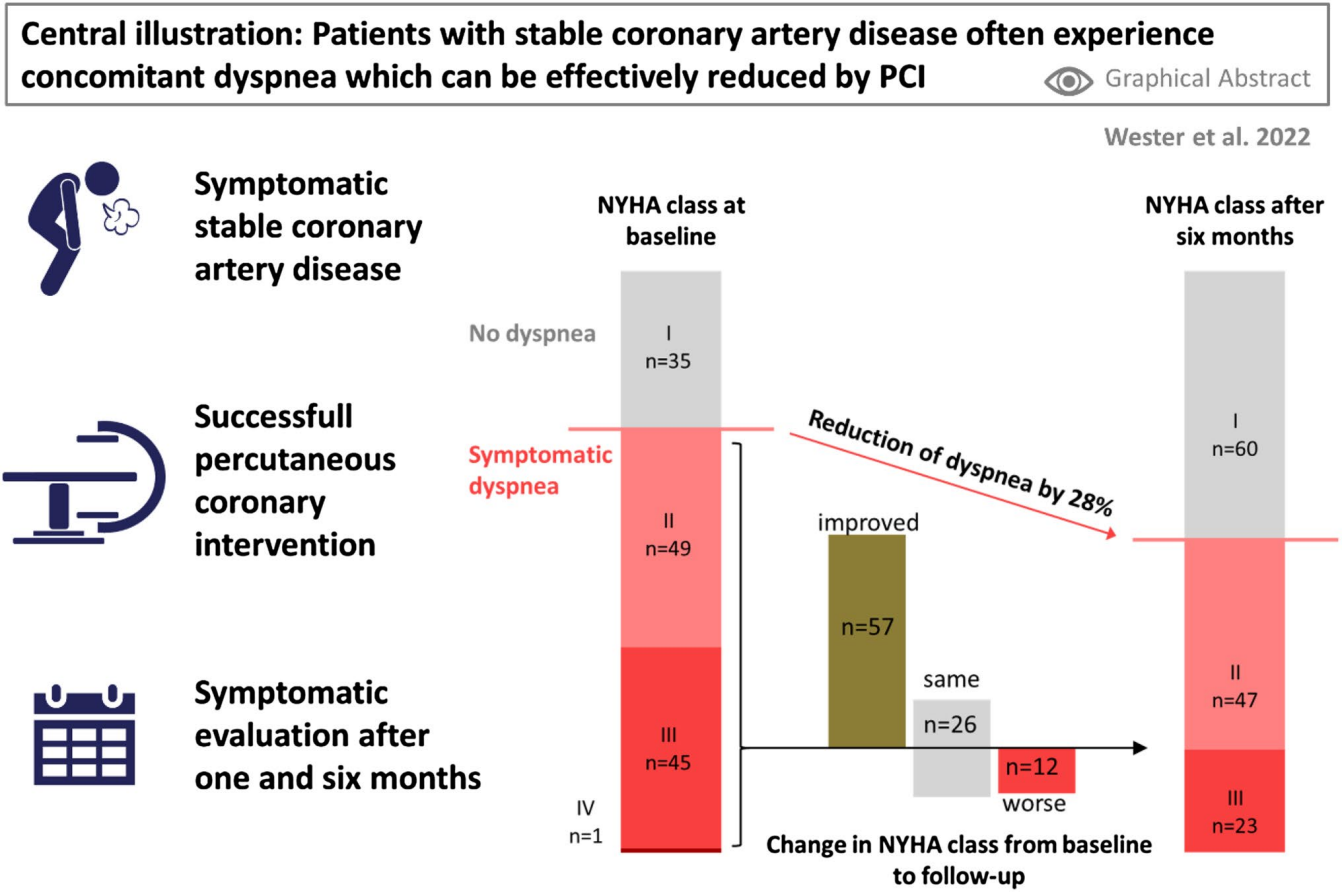

**Supplementary Information:**

The online version contains supplementary material available at 10.1007/s00392-022-02107-x.

## Introduction

Coronary artery disease (CAD) is an often debilitating disease and the symptomatic burden, including angina pectoris and dyspnea, can be accompanied by starkly impaired quality of life [1]. While a survival benefit for an invasive treatment strategy i.e., percutaneous coronary intervention (PCI) compared to medical treatment is under debate, a main therapeutic objective is a reduction of symptomatic burden [1]. Most investigations and reviews focus on angina pectoris as the main symptom of stable CAD; however, dyspnea is very common in these patients and certainly warrants more attention.

The EXCEL trial found that dyspnea was present in 73% of patients with left main coronary artery disease [2] and Quintar et al. reported that 81% of patients with chronic total occlusion presented with dyspnea [3]. On average, patients in the ISCHEMIA trial had dyspnea at moderate exercise intensity levels (Rose Dyspnea Scale [RDS] 1.2≙ “walking up a hill”), which is remarkable given that 36% did not even have angina in the last month [4]. However, it is striking that pivotal studies in the field of interventional cardiology, such as FAME-2 [5], COURAGE [6], and ORBITA [7], do not report any data about dyspnea. The AHA and ESC guidelines on stable ischemic heart disease and chronic coronary syndrome acknowledge dyspnea as a possible ischemic equivalent and a common symptom of CAD [1, 8]. The pre-test probabilities for CAD patients presenting with dyspnea reach up to 32% in men above the age of 70 [1]. However, the effects of PCI on this important symptom remain unclear [1, 8]. Qintar et al. identified factors for the improvement of dyspnea in patients with chronic total occlusion who received successful PCI and recanalization [3]; however, chronic total occlusion only represents a special minority of stable CAD and these results may not be generalized to all patients with symptomatic stable CAD.

Dyspnea often is multi-factorial in nature and causes are manifold, including numerous organ systems [9]. Underlying diseases, especially cardiac and pulmonary, often occur simultaneously [10] which complicates a clear classification of dyspnea. However, if dyspnea improves after PCI, it may be attributed to myocardial ischemia.

Large gaps of knowledge remain regarding the extent and relevance of dyspnea as a symptom of stable CAD on total symptom burden and quality of life and its susceptibility for PCI as a common treatment strategy. We therefore analyzed the burden of dyspnea in a well-characterized patient cohort presenting with stable CAD who received PCI with special regard to changes in dyspnea 6 months after PCI. We first aimed at identifying the relevance of concomitant dyspnea on the symptomatic prognosis after PCI. Patients were classified as “concomitant dyspnea” when dyspnea did occur together with angina pectoris. We then tried to evaluate the effect of PCI on total dyspnea for which we used the NYHA classification. A NYHA class of ≥ 2 was considered symptomatic dyspnea.

## Methods

### Study population

This study was designed as a prospective randomized and controlled trial to investigate the effect of visual demonstration of successful PCI on quality of life and angina pectoris in patients with symptomatic stable CAD. The details of the study design have been published previously [11]. The primary outcome of this study was the change in quality of life as assessed with the Seattle Angina Questionnaire (SAQ) from baseline to follow-up. Secondary endpoints were changes in the other SAQ-derived scores (physical limitation, angina stability, angina frequency, treatment satisfaction) and changes in the NYHA classification. Results have been published [12].

Between April 2019 and September 2020, consecutive symptomatic patients undergoing PCI at five academic centers and large community hospitals in Germany were screened for eligibility. Main inclusion criteria were as follows: age ≥ 18 years, symptomatic CAD, CCS angina score ≥ 2, angina pectoris frequency ≥ 2/week, and successful implantation of ≥ 1 coronary artery stent, i.e., complete revascularization of the culprit lesion. Main exclusion criteria were as follows: concomitant disease that could cause dyspnea or chest pain (i.e., left ventricular ejection fraction < 35%; anemia; severe pulmonary disease; severe valvular disease); conditions that prevented sufficient understanding of the visual demonstration and explanation of the angiographic results (language barrier; impaired vision or hearing; dementia).

The study complied with the Declaration of Helsinki. Each study site obtained approval by the local ethics committee (Reference number 19-1261-101) and all patients provided written informed consent for participation. The German Clinical Trials Register registration number is DRKS00017524.

### Symptomatic evaluation of angina pectoris and dyspnea

Patient-reported angina pectoris symptom burden was evaluated using the SAQ [13] at the time of hospital admission for PCI and at the follow-up visit 6 months after the procedure. The SAQ consists of the five subscales “quality of life (disease perception),” “physical limitation,” “angina frequency,” “angina stability,” and “treatment satisfaction.” The scales range from 0 points (worst symptoms) to 100 points (no symptoms). To aid in the clinical interpretation of the SAQ scales, they may be categorized into four quartile ranges: 0–24 pts. indicate a very poor to poor health status, 25–49 pts. indicate a poor to fair health status, 50–74 pts. indicate a fair to good health status, and 75–100 pts. indicate a good to excellent health status [14].

Dyspnea was determined according to the NYHA classification pre PCI, and 6 months after PCI [15]. The prespecified categorization of patients for concomitant dyspnea with angina pectoris was performed by a trained cardiologist during the inclusion visit based on the clinical history and characteristics of the dyspnea. Basically, if dyspnea typically did occur (e.g., during physical activity) and subside concordantly with angina pectoris (e.g., with rest or nitroglycerin), we considered it to be concomitant dyspnea. Dyspnea that was most likely due to an underlying pulmonary disease was not considered to be concomitant dyspnea. As the genesis of dyspnea is often multi-factorial and thus prone to confounding by comorbidities that often also cause dyspnea, we have defined uncontrolled or severe pulmonary or valvular disease or anemia as exclusion criteria.

Patients with missing data in one of the endpoints were excluded from the respective analysis. As there was no difference between the intervention and control group regarding angina pectoris [12] and dyspnea (Supplement figure), the whole study collective was evaluated for this additional analysis of the difference in dyspnea from baseline to the 6-month follow-up.

### Statistical analysis

For the present analyses, patients of the randomized controlled trial were first divided into those with “concomitant dyspnea” and those without “concomitant dyspnea.” Categorical data are presented as absolute (*n*) and relative (%) frequencies and were compared using the Chi-Square test of independence or in case of small numbers Fisher’s exact test. Continuous data are presented by means ± standard deviation (sd) or median (interquartile range [IQR]) and were compared using students *t*-test or the Mann–Whitney *U*-test.

As it can be difficult to clearly distinguish dyspnea of cardiac origin from dyspnea due to other comorbidities in patients with stable CAD, we then analyzed the study cohort according to the NYHA class of dyspnea. NYHA class I was considered “no dyspnea” and classes II to IV were labelled “symptomatic dyspnea.” To assess the effect of PCI on overall dyspnea, we presented the number of initially symptomatic patients (NYHA class II–IV at baseline) who changed in NYHA class from baseline to the 6-month follow-up. Then, predictors for an improvement of ≥ 1 NYHA class were assessed by univariable logistic regression analyses. Thus, we excluded patients without initial dyspnea because these patients could not further improve after PCI.

As choosing variables for a multivariable logistic regression model is ambiguous, we calculated two different models. Model 1 includes all variables with *p ≤ *0.1 in the univariate analysis. Model 2 includes clinically relevant variables, which were chosen based on physiologic considerations. Odds ratios (OR) and corresponding 95% confidence intervals are presented as effect estimates.

Statistical significance was defined as *p ≤ *0.05. Statistical analysis was performed using SPSS (SPSS Statistics for Windows, Version 26.0 Armonk, NY: IBM Corp.) and GraphPad Prism (Version 6.01 for Windows, GraphPad Software, La Jolla California USA).

## Results

### Patient characteristics

A total of 144 patients were included in this study. Of these, 92 patients presented without concomitant dyspnea and 52 had concomitant dyspnea. Patients with concomitant dyspnea had a slightly higher body mass index (29.4 ± 4.1 kg/m^2^ vs 27.1 ± 3.9 kg/m^2^, *p = *0.001) and higher rates of renal insufficiency (9.6% vs 27.5%, *p = *0.012) and of obstructive sleep apnea syndrome (0% vs 10.9%, *p = *0.014) than patients without concomitant dyspnea. The rate for pulmonary disease was higher in patients with concomitant dyspnea (9.8% vs 1.9%) which did not reach statistical significance (*p = *0.095). There was no difference in medication before and after PCI between patients without and with concomitant dyspnea except for a higher rate of clopidogrel before PCI in patients with concomitant dyspnea. Patient characteristics are presented in Table 1 and Supplement Table 1. The prevalence of diabetes was similar in patients with typical or atypical angina pectoris (32.0% vs 27.8%, Chi-Square *p = *0.718). There was no difference in the percentage of patients with “atypical angina” between patients with and without concomitant dyspnea (14.4% vs 9.6%, *p = *0.405).

**Table 1:** Patient characteristics

	No concomitant dyspnea *n* = 92	Concomitant dyspnea *n* = 52	*p* value
Age (years), mean ± SD	70.3 ± 9.5	69.7 ± 9.6	0.721^T^
Male sex (n, %)	31 (59.6%)	67 (72.8%)	0.102^Chi^
Body mass index (m2/kg), mean ± SD	27.1 ± 3.9	29.4 ± 4.1	**0.001**^T^
Smoking status			0.107^Chi^
Never smoking (*n*, %)	28 (53.8%)	41 (44.6%)	
Currently smoking (*n*, %)	9 (17.3%)	9 (9.8%)	
Quit smoking (*n*, %)	15 (28.8%)	42 (45.7%)	
Hemoglobin (mg/dL), mean ± SD	14.2 ± 1.6	14 ± 1.8	0.633^T^
Ejection fraction (%), mean ± SD	59.9 ± 5.6	57.7 ± 7.8	0.074^T^
Systolic blood pressure (mmHg), mean ± SD	139.0 ± 19.0	135.3 ± 19.2	0.264^T^
Diastolic blood pressure (mmHg), mean ± SD	78.4 ± 11.9	74.3 ± 13.9	0.078^T^
Heart rate (1/min), mean ± SD	70.6 ± 10.9	70.6 ± 11.4	0.975^T^
CCS class			0.690^Chi^
II (*n*, %)	25 (48.1%)	37 (40.7%)	
III (*n*, %)	26 (50.0%)	52 (57.1%)	
IV (*n*, %)	1 (1.9%)	2 (2.2%)	
Angina pectoris duration (months), median (q1; q3)	3.0 (1.25; 7.0)	3.0 (1.0; 6.0)	0.667^U^
Remaining stenosis (*n*, %)	11 (13.5%)	26 (23.5%)	0.324^Chi^
Total stent length (mm), mean ± SD	33.9 ± 24.6	36.9 ± 25.1	0.493^T^
Max. stent diameter (mm), mean ± SD	3.1 ± 0.5	3.2 ± 0.5	0.486^T^
Intervened vessels, mean ± SD	1.2 ± 0.5	1.2 ± 0.5	0.346^T^
Number of stents, mean ± SD	1.8 ± 1.3	1.9 ± 1.1	0.523^T^
Diabetes mellitus (*n*, %)	16 (30.8%)	29 (31.5%)	0.925^Chi^
Arterial hypertension (*n*, %)	44 (84.3%)	82 (89.1%)	0.431^Chi^
Hyperlipidemia (*n*, %)	37 (71.2%)	64 (69.6%)	0.841^Chi^
Atrial fibrillation (*n*, %)	2 (3.8%)	12 (13.0%)	0.074^Chi^
Heart failure (*n*, %)	3 (5.8%)	9 (9.8%)	0.537^F^
Cerebrovascular disease (*n*, %)	6 (11.5%)	7 (7.6%)	0.547^F^
Coronary artery bypass grafting (*n*, %)	7 (13.5%)	14 (15.2%)	0.774^Chi^
Renal insufficiency (*n*, %)	5 (9.6%)	25 (27.5%)	**0.012**^Chi^
Peripheral artery disease (*n*, %)	2 (3.8%)	8 (8.7%)	0.330^F^
Psychiatric disorder (*n*, %)	2 (3.8%)	6 (6.5%)	0.711^F^
Pulmonary disease (*n*, %)	1 (1.9%)	9 (9.8%)	0.095^F^
Obstructive sleep apnea syndrome (*n*, %)	0 (0%)	10 (10.9%)	**0.014**^F^

Bold values signify statistical significance. *p ≤ *0.05 calculated with the Student’s *t*-test (*T*), Mann–Whitney *U*-test (*U*), Chi-Square test (Chi), or Fisher’s exact test (*F*)

*CCS class* Canadian Cardiovascular Society angina class; *IQR* interquartile range; *SD* standard deviation

### Differences in angina symptoms and quality of life in patients with and without concomitant dyspnea

**Fig. 1:**
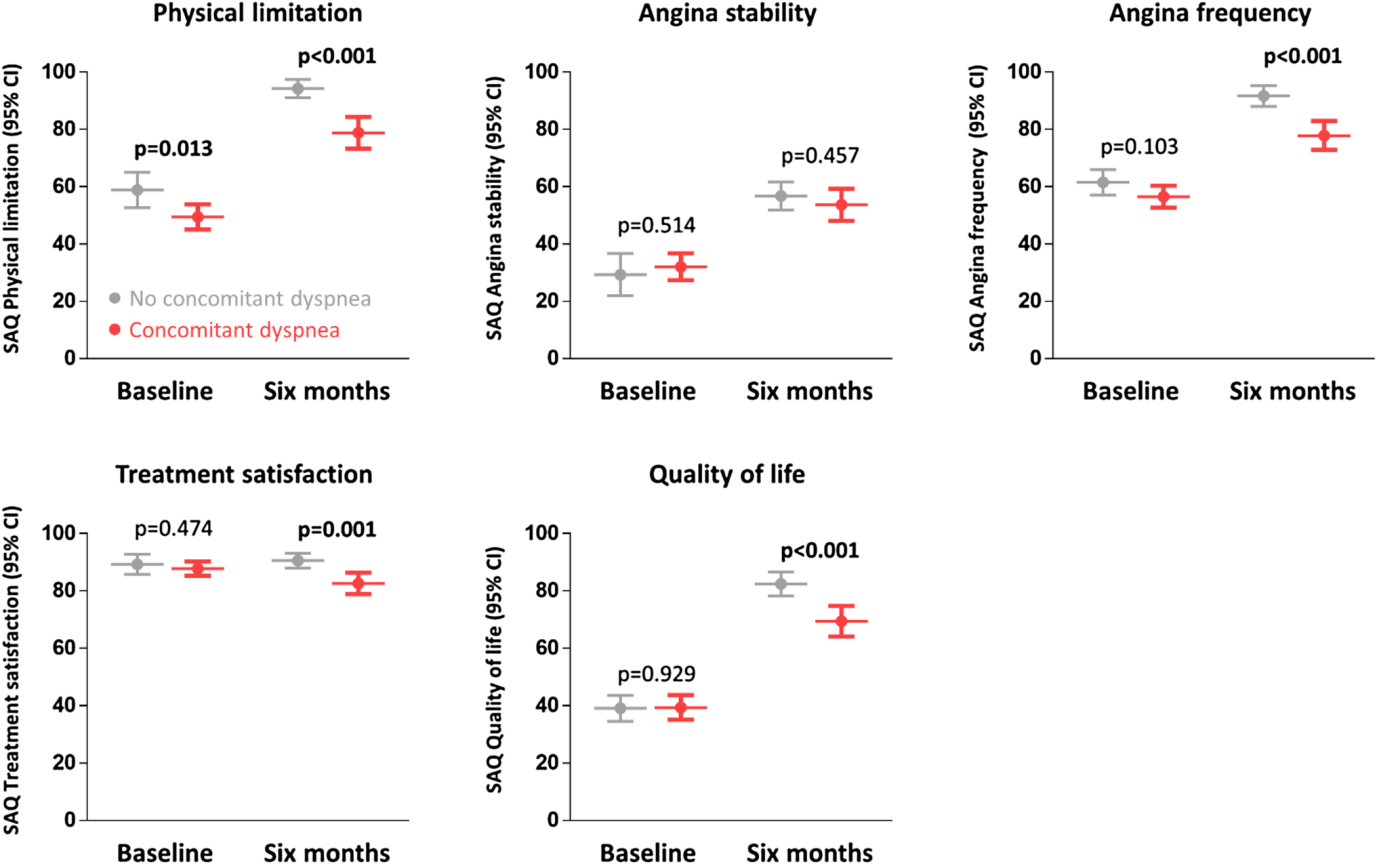
Differences in angina symptoms and quality of life in patients presenting with and without dyspnea. PCI led to a marked relief in symptomatic burden. Patients who presented with concomitant dyspnea had significant less improvement. Presented are mean (± 95% confidence interval) values for SAQ subscales at baseline and 6 months after PCI for patients presenting with (red) and without (gray) dyspnea. p-values are two-sided Student’s *t*-tests between groups at both time points. See Supplemental Table 2 for exact data

Angina pectoris symptom burden was evaluated using the SAQ subscales. At baseline, patients with concomitant dyspnea had a worse physical limitation score (49.5 ± 21.0 vs 58.9 ± 22.0, *p = *0.013) than patients without concomitant dyspnea. There was no difference between the two groups regarding angina stability (32.1 ± 22.6 vs 29.3 ± 26.5, *p = *0.514), angina frequency (56.5 ± 18.5 vs 61.5 ± 15.9, *p = *0.103), or quality of life (39.4 ± 20.8 vs 39.1 ± 16.3, *p = *0.929) at baseline (Fig. 1, Supplemental Table 2). Both groups without and with concomitant dyspnea improved strongly after PCI (Fig. 1, Supplemental Table 2). However, patients with concomitant dyspnea had worse scores than patients without concomitant dyspnea 6 months after PCI for physical limitation (78.9 ± 25.0 vs 94.3 ± 10.6, *p < *0.001), angina frequency (77.9 ± 22.8 vs 91.7 ± 12.4, *p < *0.001), and quality of life (69.4 ± 24.1 vs 82.5 ± 14.4, *p < *0.001) which are clinically and statistically highly significant.

### Effect of PCI on dyspnea (NYHA class)

It can be challenging to clearly distinguish the source of dyspnea. To account for this, we also analyzed the effect of PCI on dyspnea irrespective of cause. Therefore, dyspnea was categorized according to the NYHA scale. Complete data sets, including NYHA class at baseline and after 6 months, were available for 130 patients. There was no difference in baseline characteristics between patients with complete and incomplete data (Supplement Table 3). At baseline, 95 of 130 (73%) patients had dyspnea equal to or above NYHA class II. Six months after PCI, this number was reduced to 70 of 130 (54%). The number of patients with a NYHA class ≥ 3, representing severe impairment, was reduced from 46 (35%) to 23 (18%).

**Fig. 2:**
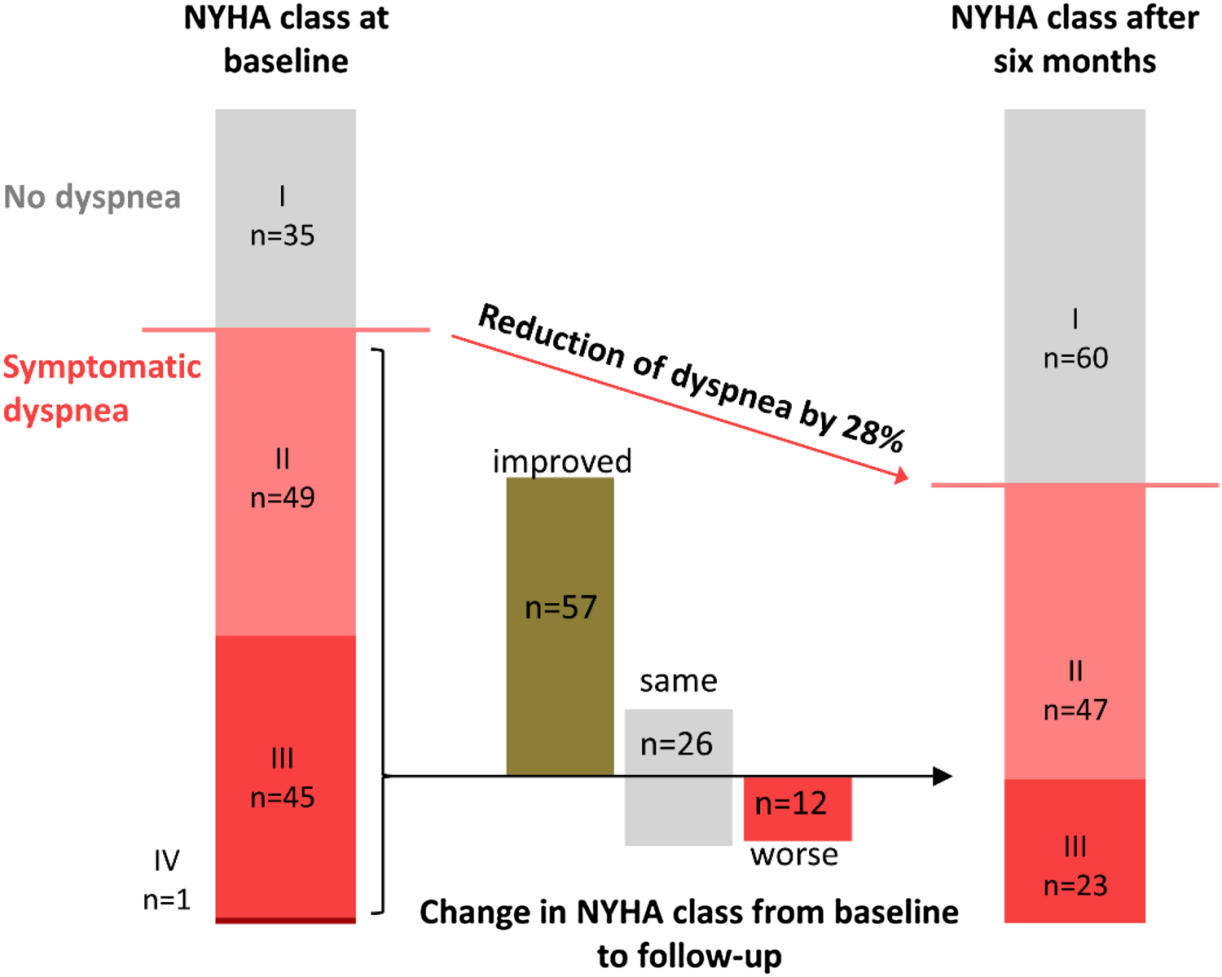
Distribution of dyspnea NYHA classes at baseline and 6 months after PCI. Overall, PCI did effectively relief dyspnea. The majority of patients experienced an improvement in NYHA class 6 months after PCI. Presented is the distribution of NYHA classes of all 138 patients with complete follow-up data after 6 months (large columns). The inlay presents the number of initially symptomatic patients (NYHA class ≥ 2) who experienced a change in NYHA class, whereas green signifies an improved, gray the same, and red a worse NYHA class at follow-up compared to baseline. See Supplemental figure for exact data

Of the 95 patients who initially presented with dyspnea, 57 (60%) improved in NYHA class, 26 (27%) did not change, and 12 (13%) deteriorated 6 months after PCI (Fig. 2). Of the 35 patients without initial dyspnea, 26 (74%) did not change and 9 (26%) deteriorated 6 months after PCI. Univariate logistic regression yielded statistically significant results for improved NYHA class for patients with a higher SAQ score for angina frequency (high values signify a lower frequency; B = 1.035 [1.010; 1.061], *p = *0.007) and for patients with atypical angina pectoris (*B* = 5.302 [1.115; 25.214], *p = *0.036). Patients with diabetes had a lower chance of improved dyspnea (*B* = 0.264 [0.107; 0.653], *p = *0.004). A multivariable regression analysis showed that diabetes mellitus and atypical angina pectoris were associated with a lower probability of reduced dyspnea independently from relevant comorbidities. The presence of concomitant dyspnea also reduced the probability of improvement in dyspnea; however, this slightly missed statistical significance (univariable regression analysis: *p = *0.058, multivariable regression analysis: *p = *0.057).

Patient characteristics, such as age (*p = *0.060) or body mass index (*p = *0.957), CAD characteristics, such as multivessel disease (*p = *0.122) or previous PCI (*p = *0.072), PCI characteristics, such as stent diameter (*p = *0.742) or number of implanted stents (*p = *0.819), or comorbidities, such as pulmonary disease (*p = *0.215) did not show a significant influence on the probability of improved NYHA class 6 months after PCI (Table 2). The study intervention of the prospective, randomized, and controlled PLA-pCi-EBO-trial which consisted of the demonstration of pre- and post-PCI angiograms to the patient did not have an effect on the probability of improved NYHA class (*p = *0.799, Table 2).

**Table 2:** Predictors for improvement of NYHA class 6 months after PCI

*n* = 93	Univariable logistic regression analysis		Multivariable logistic regression analysis			
			Model 1		Model 2	
Independent variables	OR (95% CI)	*p* value	OR (95% CI)	*p* value	*B* (95% CI)	*p* value
Diabetes	0.264 (0.107; 0.653)	**0.004**	0.265 (0.092; 0.767)	**0.014**		
Atypical angina pectoris	5.302 (1.115; 25.214)	**0.036**	5.645 (0.948; 33.605)	0.057	6.212 (1.184; 32.588)	**0.031**
Angina frequency (SAQ score)	1.035 (1.010; 1.061)	**0.007**	1.025 (0.995; 1.056)	0.108		
Physical limitation (SAQ score)	1.018 (0.998; 1.039)	**0.076**	1.010 (0.983; 1.037)	0.478		
Age (years)	0.955 (0.910; 1.002)	0.060	0.970 (0.914; 1.029)	0.310	0.975 (0.922; 1.030)	0.363
Concomitant dyspnea	0.220 (0.046; 1.054)	0.058	0.169 (0.027; 1.058)	0.057		
Previous PCI	0.469 (0.205; 1.071)	0.072	1.279 (0.423; 3.869)	0.663		
Renal insufficiency	0.407 (0.163; 1.021)	0.055	0.423 (0.126; 1.421)	0.164		
Hemoglobin (mg/dL)	1.125 (0.900; 1.405)	0.301			0.999 (0.768; 1.300)	0.994
Pulmonary disease	0.389 (0.087; 1.731)	0.215			0.327 (0.061; 1.767)	0.194
Coronary multivessel disease	0.387 (0.116; 1.291)	0.122			0.441 (0.115; 1.698)	0.234
Male sex	1.508 (0.627; 3.625)	0.359				
Body mass index (kg/m^2^)	0.997 (0.904; 1.101)	0.957				
Never smoking	1.100 (0.489; 2.476)	0.818				
Remaining stenosis > 70%	0.966 (0.483; 1.932)	0.923				
Stent diameter (mm)	0.875 (0.395; 1.938)	0.742				
Number of implanted stents	0.959 (0.669; 1.375)	0.819				
Previous myocardial infarction	0.811 (0.337; 1.951)	0.639				
Arterial hypertension	1.469 (0.411; 5.259)	0.554				
Atrial fibrillation	0.681 (0.190; 2.436)	0.554				
Heart failure	0.865 (0.217; 3.446)	0.838				
Peripheral artery disease	0.255 (0.047; 1.385)	0.113				
Obstructive sleep apnea	1.727 (0.418; 7.127)	0.450				
Image intervention group	1.111 (0.495; 2.495)	0.799				

Multivariable logistic regression analysis model 1 includes all independent variables with *p ≤ *0.1. Model 2 includes clinically relevant variables based on physiological considerations. Bold values signify statistical significance *p ≤ *0.05

*OR* Odd’s ratio; *PCI* percutaneous coronary intervention; *SAQ* Seattle Angina Questionnaire

## Discussion

### Worse symptomatic outcome after PCI in patients presenting with dyspnea

Dyspnea and angina pectoris correlate in patients with stable CAD. It is, however, not clear what the presence of dyspnea in addition to angina pectoris means for the symptomatic response after PCI. Patients who presented with concomitant dyspnea had a slightly higher body mass index (29 vs 27 kg/m^2^) and a higher rate of renal insufficiency (28% vs 10%) than patients without dyspnea. The prevalence of comorbidities, which could be associated with dyspnea – such as pulmonary disease, heart failure, or atrial fibrillation – was numerically higher in the dyspnea group. However, this was not statistically significant, which may in part be attributable to the small total number of these comorbidities. There was only a significant difference for a higher rate of obstructive sleep apnea (11% vs 0%) in the dyspnea group. It has to be noted that severe or uncontrolled pulmonary or valvular disease or relevant anemia were exclusion criteria for this study.

Chronic obstructive pulmonary disease (COPD) and CAD share smoking as a major risk factor and often occur in the same patients which may confound our findings as we did not specifically evaluate patients without known COPD for pulmonary disease. Jönsson et al. evaluated patients who were referred for further testing because of chest discomfort or dyspnea [16]. This cohort is very similar to our cohort regarding age, sex, smoking status, and body mass index. Even though COPD and ischemic heart disease were common in these patients, only a small fraction of 7% presented with both. Thus, it may be concluded that it is highly unlikely that a large proportion in our cohort has unidentified COPD as a cause for dyspnea.

Before PCI, patients with dyspnea experienced more physical limitation due to their CAD. Interestingly, the measure for quality of life did not differ between groups. There was no difference for angina stability or angina frequency. These findings suggest that there is no strong difference between patients who present with stable CAD and concomitant dyspnea or without dyspnea at baseline. However, it is striking that patients with initial dyspnea responded worse to PCI than patients without. Six months after PCI, patients with and without initial dyspnea have improved physical limitation, angina stability, angina frequency, and quality of life. However, patients with initial concomitant dyspnea have a higher angina frequency, more physical limitation, and lower quality of life.

### PCI effectively reduces dyspnea

Myocardial ischemia often manifests as dyspnea [4, 17]. Our findings are in accordance with that as 74% of the patients (108 of 144) presented with dyspnea of NYHA class II or worse. It is difficult to clearly establish the etiology of dyspnea and to differentiate between mostly pulmonary or ischemic causes especially in daily clinical routine. As dyspnea is an apparently highly relevant and possibly underrated symptom of CAD, these methodological difficulties should not prevent a detailed analysis of the connection between dyspnea and CAD and especially dyspnea’s susceptibility to PCI.

Despite being a frequent and relevant symptom of CAD [4, 17], dyspnea is often not investigated in studies evaluating the effects of PCI in coronary artery disease [5–7]. The studies that do report the effect of PCI on dyspnea included patients who received PCI for mixed indications, ranging from NSTEMI to stable CAD. The FREEDOM-trial investigated the symptomatic effect of PCI on patients with diabetes mellitus and multivessel disease, the percentage of patients with dyspnea decreased from 71% at baseline to 35% at the 6-month follow-up [18] which is in line with our findings. STEMI was an exclusion criterion for the FREEDOM trial. The indication for PCI (i.e., rates for NSTEMI, unstable angina pectoris, stable CAD) was not reported [18]. The patients were less symptomatic than our cohort as only one-third had at least weekly angina pectoris. Our findings show that a reduction of approximately one NYHA class might be expected after PCI in patients with stable CAD. This is in accordance with other studies that report a similar reduction in NYHA class for patients presenting with NSTEMI, unstable CAD, or stable CAD [4, 17]. The study by Yang et al. included 52% patients with unstable CAD [17] and the ISCHEMIA trial included 80% of patients with monthly or less angina pectoris [4]. In contrast to these populations, we concentrated on patients with well-characterized symptomatic stable coronary disease. The prognostic value of PCI is less clear in this cohort than in patients with unstable CAD and thus the goal of improving symptom burden and quality of life becomes relatively more important. It is important to note that our study did not include a sham control as, for example, the seminal ORBITA trial [7] and that thus the symptomatic improvement that occurred cannot unambiguously be attributed to the PCI, especially when considering the large placebo effect of PCI as ORBITA has demonstrated.

The exclusion criteria that we employed limit the generalizability of our results to a specific patient population, i.e., patients with symptomatic CAD and without significant comorbidities. However, this group is numerically large and highly relevant in daily clinical practice. Our results provide valuable insights for these patients.

### Predictors for improvement of dyspnea

We found that the presence of atypical angina pectoris symptoms increases, and that diabetes reduces the probability of less dyspnea 6 months after PCI. Data about predictors for changes of dyspnea after PCI are sparse. Qintar et al. investigated the effect of PCI in patients with chronic total occlusion on dyspnea [3]. They found that predictors for improvement of dyspnea 1 month after PCI and recanalization were a higher baseline dyspnea score, chronic lung disease, lower levels of depression, and higher hemoglobin levels. Diabetes did not show statistical significance. Chronic total occlusion is a peculiar subgroup of stable CAD and results obtained from this population might not be generalizable to all patients with stable CAD, which could explain the differences to our findings. However, it is interesting that neither in that cohort nor in our study, smoking, sex, age, or body mass index had an influence on the probability of dyspnea improvement. There are sparse data on predictors of symptomatic improvement after PCI, especially regarding dyspnea. Two studies assessed predictors for the improvement in quality of life after PCI. Spertus et al. found that in an univariate regression model, higher age was associated with better improvements in quality of life [19]. Quadros et al. found that in a multivariable regression model, male gender was associated with less improvement in quality of life [20]. Both studies found that the baseline health status as assessed by the SAQ was a very strong predictor of improvements in quality of life: Patients with worse scores experience more improvement after PCI, which is natural as patients with a high baseline score do not have the potential for extensive further improvement.

The finding that “atypical angina” is a predictor for better reduction of dyspnea is unexpected as clinical prejudice would conclude that these patients tend to be especially difficult to effectively treat. The prevalence of diabetes was similar in patients with typical or atypical angina pectoris; thus, diabetic polyneuropathy does not seem to be relevant in this context. We did not routinely assess microvascular dysfunction which could help differentiate the physiological differences between patients with typical and atypical angina. Furthermore, there was no difference in the percentage of patients with “atypical angina” between patients with and without concomitant dyspnea (14.4% vs 9.6%, *p = *0.405). The design and patient number of our trial do not allow for more detailed analysis of this certainly important topic and further specific studies are warranted.

There is a lack of studies aiming to identify clinical predictors for symptomatic improvement following PCI. However, this knowledge could substantially improve clinical practice by aiding in the selection of the best treatment strategy for each individual patient. In our sample, we found a negative association of diabetes with improved NYHA class after PCI, whereas atypical angina pectoris and a lower angina frequency showed a positive correlation. Equally important is the finding that many factors, which might be intuitively associated with symptomatic outcome, showed no significant relationship in our sample. These include for example age, body mass index, presence of multivessel disease, previous PCI, stent diameter, number of implanted stents, or pulmonary disease. Our findings thus add information to this field; however, further research is definitely warranted.

### Limitations

This current analysis is a secondary analysis of a randomized controlled trial, which was not powered for the present analysis strategy; generalization is therefore limited. As there was no sham control for PCI, it is not possible to attribute all improvements in symptomatic outcome after PCI exclusively to the PCI. The clinical rating of concomitant cardiac dyspnea is complex in clinical practice and some patients might have been misclassified, but it was investigated carefully by experienced cardiologists in the most appropriate manner. We used the NYHA dyspnea scale to assess dyspnea which is a generally accepted and sufficiently good tool. However, general limitations regarding clinical scales in contrast to objectifiable physiological (e.g., echocardiography) or functional testing (e.g., 6-min walking test, treadmill exercise test) regarding inaccuracy and intra- as well as interpersonal variation do also apply to the NYHA dyspnea scale.

### Conclusions

Dyspnea is often underappreciated in the context of stable CAD and data on the occurrence of dyspnea following PCI is scarce or totally lacking. We examined a very well-characterized patient cohort and found that dyspnea is a frequent and relevant symptom in patients with stable CAD. The majority of patients in our study initially presented with angina pectoris and dyspnea. We demonstrate that angina burden and quality of life of patients with concomitant dyspnea responded worse to PCI than in patients without. However, and most importantly, both angina pectoris and dyspnea can be treated effectively with PCI and symptom burden for both was significantly lower 6 months after PCI. These are important observations that should be taken into consideration when evaluating and treating patients with stable CAD and concomitant dyspnea.

## Supplementary Information

Below is the link to the electronic supplementary material.

Supplementary file1 (DOCX 72 kb)

## Data Availability

The data underlying this article will be shared on reasonable request to the corresponding author.

## Abbreviations

CAD: Coronary artery disease
CCS angina score: Canadian cardiovascular society angina grading scale
COPD: Chronic obstructive pulmonary disease
PCI: Percutaneous coronary intervention
SAQ: Seattle Angina Questionnaire
