# Supplementary material for: Effects of percutaneous coronary intervention on dyspnea in stable coronary artery disease
Source: Clin Res Cardiol. 2022 Sep 13;112(9):1194–203. doi: 10.1007/s00392-022-02107-x (PMC10449717; doi:10.1007/s00392-022-02107-x)
Supplement: Supplementary file 1 — Supplementary file1 (DOCX 72 kb) [file 392_2022_2107_MOESM1_ESM.docx]

Supplement figure. Change in dyspnea according to study groups


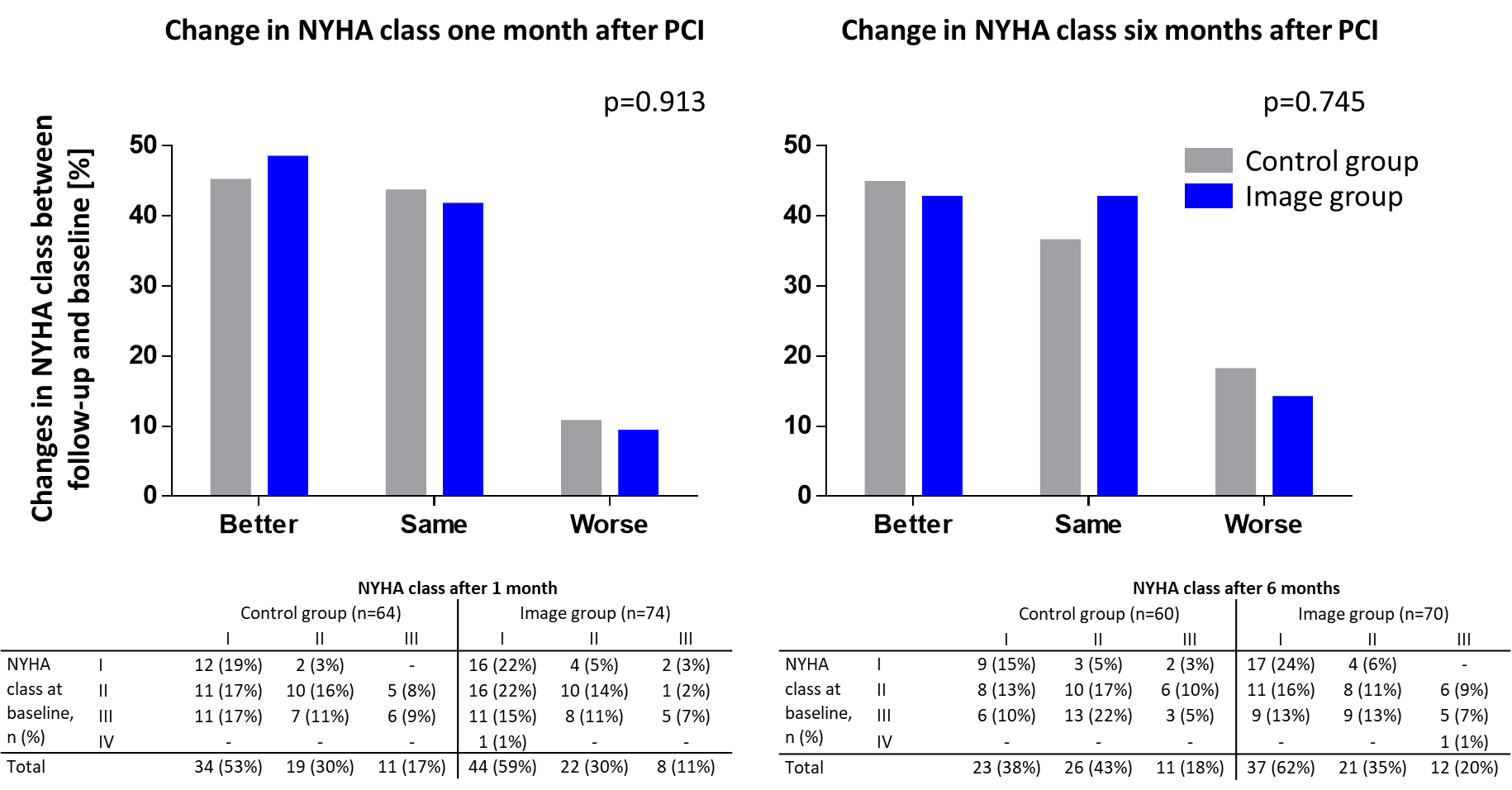


Supplement table 3: Medication before and after PCI

|  | No concomitant dyspnea | Concomitant dyspnea | p-value |  |
| --- | --- | --- | --- | --- |
| **Before PCI** |  |  |  |  |
| Aspirin | 37 (71.2%) | 58 (63.0%) | 0.324 |  |
| Clopidogrel | 0 (0%) | 16 (17.4%) | **0.001** |  |
| Ticagrelor | 1 (1.9%) | 4 (4.3%) | 0.445 |  |
| Prasugrel | 1 (1.9%) | 1 (1.1%) | 0.680 |  |
| Vit K antagonist | 0 (0%) | 3 (3.3%) | 0.188 |  |
| DOAC | 2 (3.8%) | 9 (9.8%) | 0.198 |  |
| β-blocker | 32 (61.5%) | 57 (62.0%) | 0.960 |  |
| CCB | 14 (26.9%) | 22 (23.9%) | 0.689 |  |
| Nitrates | 7 (13.5%) | 15 (16.3%) | 0.649 |  |
| Ranolazine | 2 (3.8%) | 7 (7.6%) | 0.370 |  |
| Ivabradine | 0 (0%) | 2 (2.2%) | 0.284 |  |
| Molsidomin | 2 (3.8%) | 3 (3.3%) | 0.854 |  |
| **After PCI** |  |  |  |  |
| Aspirin | 50 (96.2%) | 87 (94.6%) | 0.670 |  |
| Clopidogrel | 39 (75.0%) | 76 (82.6%) | 0.274 |  |
| Ticagrelor | 10 (19.2%) | 11 (12.0%) | 0.235 |  |
| Prasugrel | 2 (3.8%) | 5 (5.4%) | 0.670 |  |
| Vit K antagonist | 0 (0%) | 4 (4.3%) | 0.127 |  |
| DOAC | 3 (5.8%) | 10 (10.9%) | 0.305 |  |
| β-blocker | 33 (63.5%) | 59 (64.1%) | 0.936 |  |
| CCB | 18 (34.6%) | 25 (27.2%) | 0.349 |  |
| Nitrates | 2 (3.8%) | 7 (7.6%) | 0.370 |  |
| Ranolazine | 2 (3.8%) | 5 (5.4%) | 0.670 |  |
| Ivabradine | 1 (1.9%) | 2 (2.2%) | 0.919 |  |
| Molsidomin | 1 (1.9%) | 2 (2.2%) | 0.919 |  |
| Bold values signify statistical significance. p≤0.05 calculated with the Chi-Square-test.  CCB – calcium channel blocker; DOAC – direct-acting oral anti-coagulant; PCI – percutaneous coronary intervention | | | | |

Supplement table 2: Exact values for SAQ subscales at baseline and six months after PCI.

|  | **SAQ Physical limitation** | |  | **SAQ Angina stability** | |  | **SAQ Angina frequency** | |  | **SAQ Treatment satisfaction** | |  | **SAQ Quality of life** | |  |
| --- | --- | --- | --- | --- | --- | --- | --- | --- | --- | --- | --- | --- | --- | --- | --- |
|  | Baseline | Six-month follow-up |  | Baseline | Six-month follow-up |  | Baseline | Six-month follow-up |  | Baseline | Six-month follow-up |  | Baseline | Six-month follow-up |  |
| No concomitant dyspnea | 58.9±22.0 | 94.3±10.6 | p<0.001 | 29.3±26.5 | 56.8±16.9 | p<0.001 | 61.5±15.9 | 91.7±12.4 | p<0.001 | 89.3±12.6 | 90.6±8.8 | p=0.549 | 39.1±16.3 | 82.5±14.4 | p<0.001 |
| Concomitant dyspnea | 49.5±21.0 | 78.9±25.0 | p<0.001 | 32.1±22.6 | 53.7±25.3 | p<0.001 | 56.5±18.5 | 77.9±22.8 | p<0.001 | 87.8±12.1 | 82.6±16.8 | p=0.021 | 39.4±20.8 | 69.4±24.1 | p<0.001 |
|  | p=0.013 | p<0.001 |  | p=0. 514 | p=0.457 |  | p=0.103 | p<0.001 |  | p=0.474 | p=0.001 |  | p=0.929 | p<0.001 |  |

Supplement table 2: Baseline characteristics for patients with and without complete NYHA data sets

|  | Excluded in analysis (incomplete six month follow-up)  n=14 | Included in analysis  (complete six-month follow-up)  n=130 | p-Value |
| --- | --- | --- | --- |
| Age (years), mean±SD | 65.5±9.9 | 70.4±9.4 | 0.068^T^ |
| Male sex (n, %) | 9 (64.3%) | 89 (67.9%) | 0.771^F^ |
| Body mass index (m2/kg), mean±SD | 28.3±5.0 | 28.6±4.1 | 0.739^T^ |
| Smoking status |  |  | 0.606^Chi^ |
| Never smoking (n, %) | 5 (35.7%) | 65 (49.6%) |  |
| Currently smoking (n, %) | 2 (14.3%) | 16 (12.2%) |  |
| Quit smoking (n, %) | 7 (50.0%) | 50 (38.2%) |  |
| Hemoglobin (mg/dL), mean±SD | 14.1±1.5 | 13.1±1.7 | 0.974^T^ |
| Ejection fraction (%), mean±SD | 56.8±7.7 | 58.8±7.1 | 0.341^T^ |
| Systolic blood pressure (mmHg), mean±SD | 127.7±14.0 | 137.6±19.4 | 0.065^T^ |
| Diastolic blood pressure (mmHg), mean±SD | 70.4±9.4 | 76.4±13.6 | 0.111^T^ |
| Heart rate (1/min), mean±SD | 67.5±13.1 | 71.0±11.0 | 0.263^T^ |
| CCS class |  |  | 0.300^Chi^ |
| II (n, %) | 7 (50.0%) | 56 (43.1%) |  |
| III (n, %) | 6 (42.9%) | 72 (55.4%) |  |
| IV (n, %) | 1 (7.1%) | 2 (1.5%) |  |
| Angina pectoris duration (months), median (q1; q3) | 3.0 (1.0; 5.0) | 3.0 (1.0; 6.0) | 0.534^U^ |
| Remaining stenosis (n, %) | 2 (15.4%) | 35 (27.4%) | 0.431^Chi^ |
| Total stent length (mm), mean±SD | 25.3±16.5 | 36.7±25.3 | 0.128^T^ |
| Max. stent diameter (mm), mean±SD | 3.0±0.6 | 3.2±0.5 | 0.190^T^ |
| Intervened vessels, mean±SD | 1.0±0.0 | 1.2±0.5 | 0.001^T^ |
| Number of stents, mean±SD | 1.4±0.5 | 1.9±1.2 | 0.069^T^ |
| Diabetes mellitus (n, %) | 4 (28.6%) | 41 (31.3%) | 1.000^F^ |
| Arterial hypertension (n, %) | 11 (78.6%) | 116 (88.5%) | 0.384^F^ |
| Hyperlipidemia (n, %) | 9 (64.3%) | 93 (71.0%) | 0.759^F^ |
| Atrial fibrillation (n, %) | 1 (7.1%) | 13 (9.9%) | 1.000^F^ |
| Heart failure (n, %) | 1 (7.1%) | 11 (8.4%) | 1.000^F^ |
| Cerebrovascular disease (n, %) | 0 (0%) | 13 (9.9%) | 0.616^F^ |
| Coronary artery bypass grafting (n, %) | 2 (14.3%) | 19 (14.5%) | 1.000^F^ |
| Renal insufficiency (n, %) | 1 (7.1%) | 29 (22.3%) | 0.301^F^ |
| Peripheral artery disease (n, %) | 1 (7.1%) | 9 (6.9%) | 1.000^F^ |
| Psychiatric disorder (n, %) | 1 (7.1%) | 7 (5.3%) | 0.566^F^ |
| Pulmonary disease (n, %) | 1 (7.1%) | 9 (6.9%) | 1.000^F^ |
| Obstructive sleep apnea syndrome (n, %) | 0 (0%) | 10 (7.6%) | 0.598^F^ |
| Bold values signify statistical significance. p≤0.05 calculated with the Student’s t-test (T), Mann-Whitney-U-test (U), Chi-Square-test (Chi), or Fisher’s exact test (F). CCS class – Canadian Cardiovascular Society angina class; IQR – interquartile range; SD – standard deviation | | | |
